# Exploring Criticality as a Generic Adaptive Mechanism

**DOI:** 10.3389/fnbot.2018.00055

**Published:** 2018-10-02

**Authors:** Miguel Aguilera, Manuel G. Bedia

**Affiliations:** ^1^Information and Autonomous Systems-Research Center for Life, Mind, and Society, University of the Basque Country, Donostia, Spain; ^2^Department of Computer Science and Systems Engineering, University of Zaragoza, Zaragoza, Spain; ^3^Interactive Systems, Adaptivity, Autonomy and Cognition Lab, Aragón Institute of Engineering Research, University of Zaragoza, Zaragoza, Spain

**Keywords:** criticality, learning, boltzmann machine, Ising model, heat capacity

## Abstract

The activity of many biological and cognitive systems is not poised deep within a specific regime of activity. Instead, they operate near points of critical behavior located at the boundary between different phases. Certain authors link some of the properties of criticality with the ability of living systems to generate autonomous or intrinsically generated behavior. However, these claims remain highly speculative. In this paper, we intend to explore the connection between criticality and autonomous behavior through conceptual models that show how embodied agents may adapt themselves toward critical points. We propose to exploit maximum entropy models and their formal descriptions of indicators of criticality to present a learning model that drives generic agents toward critical points. Specifically, we derive such a learning model in an embodied Boltzmann machine by implementing a gradient ascent rule that maximizes the heat capacity of the controller in order to make the network maximally sensitive to external perturbations. We test and corroborate the model by implementing an embodied agent in the Mountain Car benchmark test, which is controlled by a Boltzmann machine that adjusts its weights according to the model. We find that the neural controller reaches an apparent point of criticality, which coincides with a transition point of the behavior of the agent between two regimes of behavior, maximizing the synergistic information between its sensors and the combination of hidden and motor neurons. Finally, we discuss the potential of our learning model to answer questions about the connection between criticality and the capabilities of living systems to autonomously generate intrinsic constraints on their behavior. We suggest that these “critical agents” are able to acquire flexible behavioral patterns that are useful for the development of successful strategies in different contexts.

## 1. Introduction

In the field of cognitive science, the interest in developing models of intrinsic motivation is unquestionable. The practical uses are related to the possibility of having more autonomous artifacts. In recent years, a significant number of models and cognitive architectures have been developed in the literature, pursuing various methods to get better intrinsically motivated machines. However, most of these studies follow *ad hoc* rules or present many conceptual weaknesses (Oudeyer and Kaplan, 2009). Therefore, it is a major research challenge to find new methods for designing intrinsically motivated systems.

One of the most intriguing intuitions in this field is the one that considers that the best way for machines to acquire skills completely on their own (and useful to pursue goals) is by exploiting the sensorimotor patterns that they create during their body-environment interactions. In this sense, they would be able to quickly construct more complex behaviors using a second level of learning from these patterns, so that they could combine typical random exploration with goal-free exploration, handling useful information obtained during their interactions with the world.

This insight, initially proposed in Juarrero (1999), is the complete opposite of the one that exists in artificial intelligence. The traditional way of dealing with notions such as motivations, autonomous goals, or intentional behavior implicitly assumes a reductionist perspective about how the mind causes behaviors. Engineers basically program “desires” within the artifacts as specific instructions that are extrinsic to them. Considering the fact that the actions generated from prior mental entities imply the existence of a “homuncular assumption” underlying every action. In this sense, intentional behavior and its causes could be better understood as a dynamical process that takes shape through the interactions between organisms and their environments (Buhrmann et al., 2013). Thus, intentionality could be described from a complex dynamical perspective that raises profound implications in relation to the notions of causation and intention in human action. This changes the view of how the intentions that “one feels” exist as independent mental events, proposing instead a new perspective where they result from the self-organizing tendencies in the human-environment system. Intentions to act, from this perspective, are best characterized as dynamical processes that are embedded in the physical and social history of a cognitive agent and are constrained to the set of limited alternatives within the self-organized space that is defined for particular agent-environment interactions. This idea has been followed by several authors and has been exploited in the field of autonomous robotics. In particular, some progresses have been made in measuring how a robot could manage the information it receives by applying information theory metrics (Der et al., 2008; Martius et al., 2013; Wissner-Gross and Freer, 2013).

In this paper, we are interested in developing models with intrinsic motivations that are generated through the exploitation of the information in sensorimotor patterns. In particular, we are interested in designing an embodied agent that generates complex behavior by adapting to operate near critical points. Criticality is a ubiquitous phenomenon in nature, both in physical and biological systems. It refers to a distinctive set of properties that are found at the boundary that separates regimes with different dynamics: the transition between an ordered phase and a disordered phase. Some of these properties include (i) power-law divergences of some quantities that are described by critical exponents and (ii) maximal sensitivity to external perturbations (Salinas, 2001a,b). With regard to our interests, it is known that self-organizing properties that allow us to characterize “modes of critical behavior” are related to different functional domains of cognitive activity (Van Orden et al., 2003, 2012; Dixon et al., 2012). This leads us to think that criticality may be functionally useful in problem solving.

Most of the systems near critical points exhibit a wide range of time scales in their dynamics, being maximally responsive to certain external signals. For a system facing a problem, critical states leave open different courses of action (configured within a global state that is acutely context sensitive) that can be simultaneously constrained in only one course of action in an effective way. Hoffmann and Payton (2018) showed that this type of self-organizing critical processes can even be used to solve optimization problems with many local minima, in a more efficient way when compared to other random search methods.

It has also been conjectured that systems that show intentional behavior should self-organize into critical states (Van Orden and Holden, 2002; Van Orden et al., 2003), but, nevertheless, the connection between self-organized criticality and intrinsically generated behavior remains highly speculative. In general, although evidence of criticality has been found using different experimental methods, the connection between these indicators and the properties of mechanistic models of critical activity is thin (Wagenmakers et al., 2012). This makes it difficult to assess the connection between criticality and other cognitive phenomena, other than at the level of pure analogy. Interestingly, in the past few years, large sets of biological data have allowed the characterization–using maximum entropy models–of how the behaviors of different biological systems (e.g., networks of neurons, antibody segments, or flocks of birds) are poised near a critical point within their parameter space (Mora and Bialek, 2011; Tkacik et al., 2015). This has been a great step toward the development of deeper theoretical principles that lie behind the behavior of biological and cognitive systems. However, going beyond the importance of these models in explaining the emergence of criticality in specific experimental data, we propose a complementary perspective to address the development of “conceptual models” to explain how organisms are driven toward critical behavior at a more abstract level and what the behavioral correlates are when the agent adapts to critical points.

With all of the above information, in this paper, we seek to develop a mechanism that combines these two concepts: criticality and models of intrinsic motivation. In the study of cognitive processes, criticality always appears to be entangled with other features of adaptive behavior (e.g., perception, prediction, learning) in agents that interact with complex environments. Here, we use conceptual modeling that allows us to study this relationship in a neutral and abstract way.

Therefore, the aim of this paper is to propose a model that is able to drive synthetic agents toward critical points to potentially clarify what the contribution of criticality is in different contexts. Instead of making assumptions about the underlying dynamics of the elements of the agent's controller or a fine-tuning of the parameters of the system, our approach makes use of concepts from statistical mechanics to exploit macroscopic variables that drive the system to transition points between qualitatively different regimes of behavior. Some authors have studied the computational capabilities of recurrent neural networks that operate near the edge of chaos, that is, the transition from ordered to chaotic dynamics (Bertschinger and Natschläger, 2004). Here, we propose a conceptual model (a Boltzmann machine) that allows us to exploit its statistical properties to derive a learning rule to drive an embodied agent toward critical points of its parameter space. Maximum entropy models have the advantage of providing a formal description of the statistical distribution of the system, which we will exploit to characterize the indices of criticality and to derive rules that maximize these indices. An abstract mechanism that drives the agents to near critical points in different scenarios may help in understanding what the contributions of criticality “by itself” are or how criticality is related to other phenomena.

The paper is organized as follows. First, we introduce a Boltzmann machine as the simplest statistical mechanics model of pairwise correlations between elements of a network and, then, derive a learning model for driving the system toward critical points. The model exploits the heat capacity of the system, a macroscopic measure that works as a proxy for criticality (when the heat capacity diverges, a Boltzmann machine is at a critical point). Consequently, we test our learning model in an embodied agent that controls a Mountain Car (a classic reinforcement learning test bed) by finding that it is able to drive both the neural controller and the behavior of the agent to a transition point in the parameter space between qualitatively different behavioral regimes. Finally, we discuss the possible applications of our model to contribute to the development of deeper principles that govern biological and cognitive systems.

## 2. Driving a neural controller toward a critical point

We propose a learning model for adjusting the parameters of a Boltzmann machine in order to drive the system near states of criticality. We take advantage of the fact that, at critical points, derivatives of thermodynamic quantities such as entropy may diverge (Mora and Bialek, 2011). An example of this is heat capacity, whose divergence is a sufficient condition for criticality (though not a necessary one). As discussed below, the heat capacity of a system is related to the derivative of the entropy of the system. If the heat capacity of the system diverges at a critical point, this means that the system is maximally sensitive to external perturbations, since very small perturbations push the system into order or disorder. We hypothesize that actively seeking to poise a system near a critical point may constitute an intrinsic mechanism to adapt to different environments and generate complex behaviors in different contexts.

We define our model as a stochastic artificial neural network (i.e., a Boltzmann machine) (Ackley et al., 1985) that follows a maximum entropy distribution:

(1) $$P(\sigma )=\frac{1}{Z}exp\left[\beta \sum_{i}h_{i}\sigma_{i}+\sum_{i<j}J_{ij}\sigma_{i}\sigma_{j}\right]$$

where the distribution follows an exponential family $P\left(\sigma \right)=\frac{1}{Z}e^{-\beta E\left(\sigma \right)}$, *Z* is a normalization value, the energy *E*(σ) of each state is defined in terms of the bias *h*_*i*_ and symmetrical couplings *J*_*ij*_ between pairs of units, and β = 1/(*Tk*_*B*_), *k*_*B*_ is Boltzmann's constant and T is the temperature of the system.

Throughout the paper, we simulated the network that updates its state by applying Glauber dynamics to all the units within the network in a sequential random order at each simulation step. Glauber dynamics define the probability of the next state of a neuron *i* as

(2) $$P(\sigma_{i}^{\prime }|\sigma )=\frac{1}{1+e^{-2\beta \sigma_{i}^{\prime }H_{i}}},\;H_{i}=h_{i}+\sum_{j}J_{ij}\sigma_{j}$$

where *s* is the state of the system at time *t* and *s*′ is the state at time *t* + 1.

In order to know if the system is near a critical point, typically, the divergence of certain quantities is measured. One of these quantities is the heat capacity of the system, which is generally defined as

(3) $$C(\sigma )=-\beta \frac{\partial S(\sigma )}{\partial \beta }=\beta^{2}\left(\langle E{\left(\sigma \right)}^{2}\rangle -{\langle E(\sigma )\rangle }^{2}\right)$$

where $S\left(\sigma \right)=-\underset{\sigma }{\sum }P\left(\sigma \right)\log\left(P\left(\sigma \right)\right)$, and the heat capacity is defined in terms of the global energy of the system $E\left(\sigma \right)=-\underset{i}{\sum }h_{i}\sigma_{i}-\underset{i<j}{\sum }J_{ij}\sigma_{i}\sigma_{j}$, making it impractical to derive learning rules based on local information. This will be important for applying our learning rule to an embodied agent, where the energy of the states of the environment is not directly accessible to the system. Instead, we can find a more tractable indicator of criticality by defining the heat capacity of the system from the conditional entropy of each neuron that depicts transitions between states. We define conditional entropy of a neuron *i* as

(4) $$\begin{array}{l} S(\sigma_{i}^{\prime }|\sigma )=-\sum_{\sigma }P(\sigma )\sum_{\sigma_{i}^{\prime }}\log(P(\sigma_{i}^{\prime }|\sigma ))\cdot P(\sigma_{i}^{\prime }|\sigma )\\ \;=-\sum_{\sigma }P(\sigma )\left(\beta H_{i}\tanh(\beta H_{i})-\log(2\cosh(\beta H_{i})\right) \end{array}$$

Thus, we define the heat capacity associated with the conditional entropy of neuron *i* as

(5) $$\begin{array}{l} C(\sigma_{i}^{\prime }|\sigma )\;=-\beta \frac{\partial S(\sigma_{i}^{\prime }|\sigma )}{\partial \beta }=\sum_{\sigma }P(\sigma )(\frac{H_{i}^{2}\beta^{2}}{\cosh{\left(\beta H_{i}\right)}^{2}}\\ \;-\beta \left(E(\sigma )-\langle E(\sigma )\rangle )(\beta H_{i}\tanh(\beta H_{i})-\log(2\cosh(\beta H_{i})\right)) \end{array}$$

which still contains terms that are dependent on the global energy of the system *E*(σ). In order to derive a learning rule based only on local information, we can introduce individual temperatures *T*_*i*_ for each neuron, which are associated with an individual inverse temperature β_*i*_. In other words, instead of modifying the temperature of the system as a whole, we introduce the possibility of modifying “individual temperatures.” We use these quantities to derive a simplified version of the heat capacity that can be computed as an average of a function that is defined only by local variables,

(6) $$\begin{array}{l} C_{i}^{\prime }\;=-\beta_{i}\frac{\partial S(\sigma^{\prime }|\sigma )}{\partial \beta_{i}}=\sum_{\sigma }P(\sigma )(\frac{H_{i}^{2}\beta_{i}^{2}}{\cosh{\left(\beta_{i}H_{i}\right)}^{2}}+\beta_{i}(\sigma_{i}H_{i}\\ -\langle \sigma_{i}H_{i}\rangle )\left(\beta_{i}H_{i}\tanh(\beta_{i}H_{i})-\log(2\cosh(\beta_{i}H_{i})\right)) \end{array}$$

and we compute the total approximated heat capacity as $C^{\prime }=\underset{i}{\sum }C_{i}^{\prime }$. Note that the properties of *C*′ and $C\left(\sigma_{i}^{\prime }|\sigma \right)$ can be different, since we are neglecting the terms that reflect global interactions, retaining only local interactions. However, we argue that the simplified *C*′ may be a suitable indicator for driving a system to a critical point.

**Figure 1:**
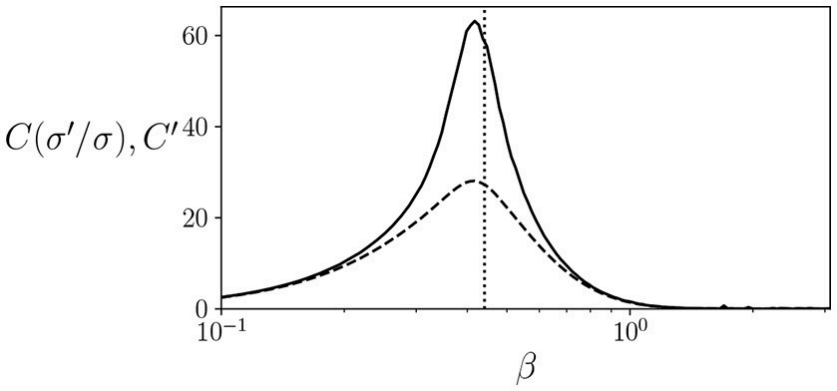
Values of $C\left(\sigma_{i}^{\prime }|\sigma \right)$ (solid line) and *C*′ (dashed line) for an 8 × 8 lattice Ising model at different temperatures. We find that both quantities show a peak around the critical temperature at $\beta =\log\left(1+\sqrt{2}\right)/2$ (dotted line).

As an example, we can compute the values of $C\left(\sigma_{i}^{\prime }|\sigma \right)$ and *C*′ for the well known Ising model in a rectangular lattice, for which the critical temperature is known to be at $T=2k_{B}/\log\left(1+\sqrt{2}\right)$ for an infinite-size system. Note that, since *C* and *C*′ can be defined as an average of the terms by multiplying *P*(σ) in equations, in practice it can be approximated by running a system for several steps and computing the mean value of these terms. We simulate an 8 × 8 periodic square lattice during 100,000 simulation steps. As seen in Figure 1, both $C\left(\sigma_{i}^{\prime }|\sigma \right)$ and *C*′ have a peak around the critical temperature, although the peak is more pronounced in the case of $C\left(\sigma_{i}^{\prime }|\sigma \right)$, showing how, at least in some cases, a peak in *C*′ can be an indicator of proximity to a critical point.

An earlier study (Aguilera and Bedia, 2018) used indirect indicators as the distribution of correlations of the system to find points of divergence of the heat capacity. Here, we try to directly maximize the heat capacity by obtaining the relation between parameter changes and the heat capacity. We define a learning rule that adjusts the values of *h*_*i*_ and *J*_*ij*_ by using a gradient ascent rule that maximizes the value of the simplified heat capacity *C*′, with the intention of driving the system to critical points that are depicted by a singularity of the heat capacity.

In order to simplify the notation, we define the quantities *F*_*i*_ = *H*_*i*_tanh(*H*_*i*_)−log(2cosh(*H*_*i*_), $G_{i}=\frac{H_{i}^{2}}{\cosh{\left(H_{i}\right)}^{2}}+\sigma_{i}H_{i}F_{i}$, and *K*_*i*_ = 〈σ_*i*_*H*_*i*_〉. Using the derivatives of the probability distribution in Equation 1, $\frac{\partial P\left(\sigma \right)}{\partial h_{i}}=\left(\sigma_{i}-\langle \sigma_{i}\rangle \right)P\left(\sigma \right)$ and $\frac{\partial P\left(\sigma \right)}{\partial J_{ij}}=\left(\sigma_{i}\sigma_{j}-\langle \sigma_{i}\sigma_{j}\rangle \right)P\left(\sigma \right)$, and the derivatives of *F*_*i*_, *G*_*i*_, and *K*_*i*_, we derive the learning rule that ascends the gradient of $C_{i}^{\prime }$ and drives the system toward critical points as

(7) $$\begin{array}{l} \frac{\partial C_{i}^{\prime }}{\partial h_{i}}=\langle \frac{\partial G_{i}}{\partial h_{i}}\rangle +\langle \sigma_{i}G_{i}\rangle -\langle \sigma_{i}\rangle \langle G_{i}\rangle -\frac{\partial K_{i}}{\partial h_{i}}\langle F_{i}\rangle -K_{i}(\langle \frac{\partial F_{i}}{\partial h_{i}}\rangle \\ +\langle \sigma_{i}F_{i}\rangle -\langle \sigma_{i}\rangle \langle F_{i}\rangle )\\ \frac{\partial C_{i}^{\prime }}{\partial J_{ij}}=\langle \frac{\partial G_{i}}{\partial J_{ij}}\rangle +\langle \sigma_{i}\sigma_{j}G_{i}\rangle -\langle \sigma_{i}\sigma_{j}\rangle \langle G_{i}\rangle -\frac{\partial K_{i}}{\partial J_{ij}}\langle F_{i}\rangle -K_{i}(\langle \frac{\partial F_{i}}{\partial J_{ij}}\rangle \\ +\langle \sigma_{i}\sigma_{j}F_{i}\rangle -\langle \sigma_{i}\sigma_{j}\rangle \langle F_{i}\rangle ) \end{array}$$

where

(8) $$\begin{array}{l} \frac{\partial F_{i}}{\partial h_{i}}=\frac{H_{i}}{\cosh{\left(H_{i}\right)}^{2}},\\ \frac{\partial F_{i}}{\partial J_{ij}}=\frac{H_{i}\sigma_{j}}{\cosh{\left(H_{i}\right)}^{2}},\\ \frac{\partial G_{i}}{\partial h_{i}}=\frac{2H_{i}(1-H_{i}\tanh(H_{i}))}{\cosh{\left(H_{i}\right)}^{2}}+\sigma_{i}F_{i}+\sigma_{i}H_{i}\frac{\partial F_{i}}{\partial h_{i}},\\ \frac{\partial G_{i}}{\partial J_{ij}}=\frac{2H_{i}\sigma_{j}(1-H_{i}\tanh(H_{i}))}{\cosh{\left(H_{i}\right)}^{2}}+\sigma_{i}\sigma_{j}F_{i}+\sigma_{i}H_{i}\frac{\partial F_{i}}{\partial J_{ij}},\\ \frac{\partial K_{i}}{\partial h_{i}}=\langle \sigma_{i}\rangle +\langle \sigma_{i}^{2}H_{i}\rangle -\langle \sigma_{i}\rangle K_{i}\\ \frac{\partial K_{i}}{\partial J_{ij}}=\langle \sigma_{i}\sigma_{j}\rangle +\langle \sigma_{i}^{2}\sigma_{j}H_{i}\rangle -\langle \sigma_{i}\sigma_{j}\rangle K_{i} \end{array}$$

In the following section, we use this learning rule to drive the neural controller of an embodied agent toward a critical point. In order to do so, we need to take into account the environment at the time of learning. If we consider two interconnected Boltzmann machines (one being the neural controller and the other being the environment), Equation 7 holds perfectly, and we could design an adaptive controller that applies the rule to the values of *i* and *j* that correspond to units of the neural controller. In our case, the environment is not composed of units of a Boltzmann machine. Instead, we connect the Boltzmann machine of the neural controller to an environment that is defined as a classic example from reinforcement learning. Therefore, our learning rule will be valid as long as the statistics of the environment can be approximated by a Boltzmann machine with a sufficiently large number of units. Luckily, Boltzmann machines are universal approximators (Montúfar, 2014).

## 3. Embodied model: mountain car

**Figure 2:**
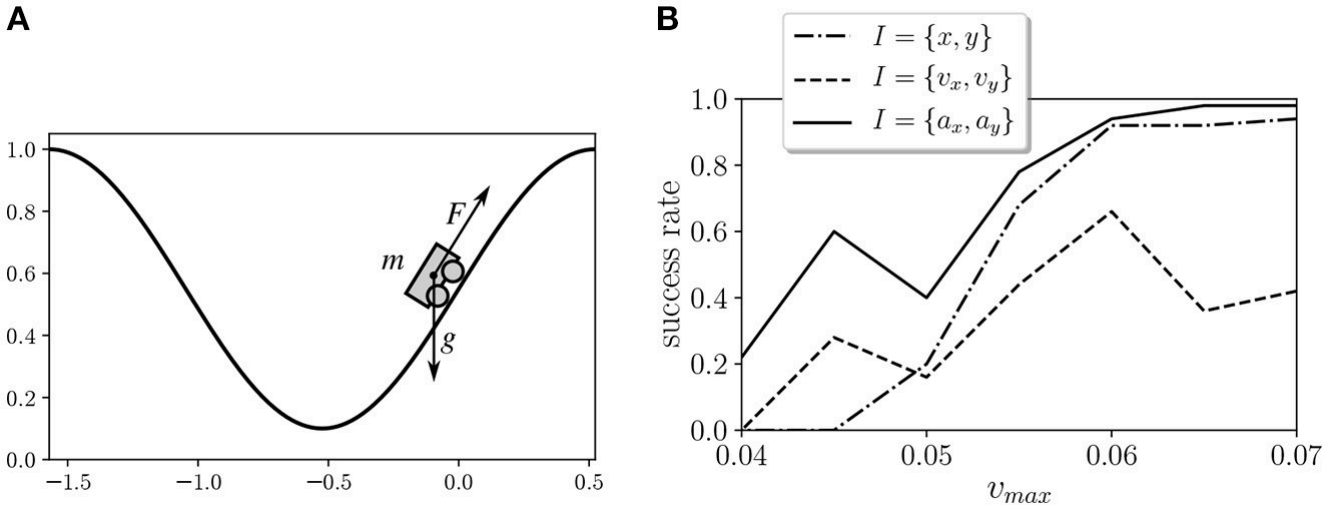
**(A)** Mountain Car environment test from the OpenAI Gym toolkit. An underpowered car that must drive up a steep hill by balancing itself to gain momentum. **(B)** Success rates of the Mountain Car environment with different kinds of sensors.

In order to evaluate the behavior of the proposed learning model, we tested it in the Mountain Car environment (Moore, 1990). This environment is a classical test bed in reinforcement learning that depicts an underpowered car that must drive up a steep hill (Figure 2). Since gravity is stronger than the car's engine, the vehicle must learn to leverage its potential energy by driving to the opposite hill before the car is able to make it to the goal at the top. We simulate the environment by using the OpenAI Gym toolkit (Brockman et al., 2016). In this environment, the horizontal position *x* of the car is limited to an interval of [−1.5π/3, 0.5π/3], and the vertical position of the car is defined as *y* = 0.5(1 + sin(3*x*)). The velocity in the horizontal axis is updated at each time step as *v*_*x*_(*t* + 1) = *v*_*x*_(*t*) + 0.001*a* − 0.0025 cos(3*x*), where *a* is the action of the motor, which can be either −1, 0, or 1. The maximum velocity of the car is limited to an absolute value of *v*_*max*_.

We defined the neural controller of the car as a fully-connected Boltzmann machine (without hidden neurons) that contains six sensors and six neurons. Initially, we tested different options of input: position, speed, and acceleration. For each input, the value is separated into its horizontal and vertical components, each input is discretized as arrays of three bits. Each sensor unit is assigned a value of 1 if its corresponding bit is active and a value of −1 otherwise. Two of the car neurons are connected to the motors, defined as *a* = 1 if both neurons are active, *a* = −1 if both neurons are inactive, and *a* = 0 otherwise.

In order to find critical points with maximum heat capacity, we propose a learning rule intended to climb the gradient defined by Equation 7 at a rate μ. Also, in order to avoid overfitting, we add an L2 regularization term λ penalizing large values of *h*_*i*_ and *J*_*ij*_ the parameters of the system. Finally, the learning rule is described as:

(9) $$\begin{array}{l} h_{i}\leftarrow h_{i}+\mu \frac{\partial C_{i}^{\prime }}{\partial h_{i}}-\lambda h_{i}\\ J_{ij}\leftarrow J_{ij}+\mu \frac{\partial C_{i}^{\prime }}{\partial J_{ij}}-\lambda J_{ij} \end{array}$$

where μ = 0.02, λ = 0.002, and $\frac{\partial C_{i}^{\prime }}{\partial h_{i}}$ and $\frac{\partial C_{i}^{\prime }}{\partial J_{ij}}$ are the result of Equation 7. The rule is applied to 20 different agents. Agents are initialized in the starting random position of the environment. Hidden and motor neurons are randomized, and the initial parameters *h* and *J* are sampled from a uniform random interval [−0.01, 0.01]. The agents are simulated for 1, 000 trials of 5, 000 steps, applying Equation 7 at the end of the trial for computing the values of $\frac{\partial C_{i}^{\prime }}{\partial h_{i}}$ and $\frac{\partial C_{i}^{\prime }}{\partial J_{ij}}$. Note that the agents are not reset at the end of the trial. After training, the values of *h*_*i*_ and *J*_*ij*_ are kept fixed for the rest of the analysis described in the paper.

We tested different types of inputs and values of *v*_*max*_. The inputs tested were 1) the horizontal position and vertical position of the car *I* = {*x, y*}, 2) the horizontal speed and vertical speed of the car *I* = {*v*_*x*_, *v*_*y*_}, and 3) the horizontal acceleration and vertical acceleration of the car *I* = {*a*_*x*_, *a*_*y*_}. In all cases, horizontal and vertical values are discretized as arrays of three bits and are fed to the six sensor units. We tested seven values of *v*_*max*_ in the range [0.04, 0.07] for the three types of inputs and the 20 agents, and we measured the success of the agents as their ability to reach the top of the agents in a trial of 50,000 steps after training (Figure 2B). In order to select a case where the task is feasible but not too easy, we chose *I* = {*a*_*x*_, *a*_*y*_} and *v*_*max*_ = 0.045 for the experiments described below. The experimental results correspond to the 20 agents trained for this configuration.

## 4. Results

In this section, we analyze the neural controller and the behavioral patterns of the agents in relation to the possibilities of their parameter space. In order to compare the agents with other behavioral possibilities, we explore the parameter space by changing the parameter β. Since the temperature of the model has no physical significance, modifying the value of β is equivalent to a global rescaling of the parameters of the agent that transforms *h*_*i*_ ← β · *h*_*j*_ and *J*_*ij*_ ← β · *J*_*ij*_, thus, exploring the parameter space along one specific direction. For 21 values of β that are logarithmically distributed in the interval [10^−1^, 10^1^], we compute 20 agents for a trial of 10^6^ simulation steps, after starting the agents from a random initial position (i.e., *x* in an interval [0.4, 0.6]) and a run of 10^4^ simulation steps to avoid the initial transient. We use the results of these simulations for all the calculations in this section.

### 4.1. Signatures of criticality in the neural controller

Firstly, we test whether the trained agents show signatures of critical behavior, looking for a Zipf's law in the probability distribution of the states of the neural controller and a peak in its heat capacity. In order to test that the criticality arises from the agent's configuration and not just from dynamics of the task, we compared the results of the trained agents with the 20 agents trained for maximizing the success in the task. In order to do so, we trained agents with a similar network by using a microbial genetic algorithm (Harvey, 2009) that maximizes the number of times an agent is able to climb the mountain during the 5,000 steps (reseting the agent after each climb). By counting the occurrence of each possible state of the 12 neurons of the agents (including sensor, hidden, and motor neurons), we can compute the probability distribution of the Boltzmann machine *P*(σ).

**Figure 3:**
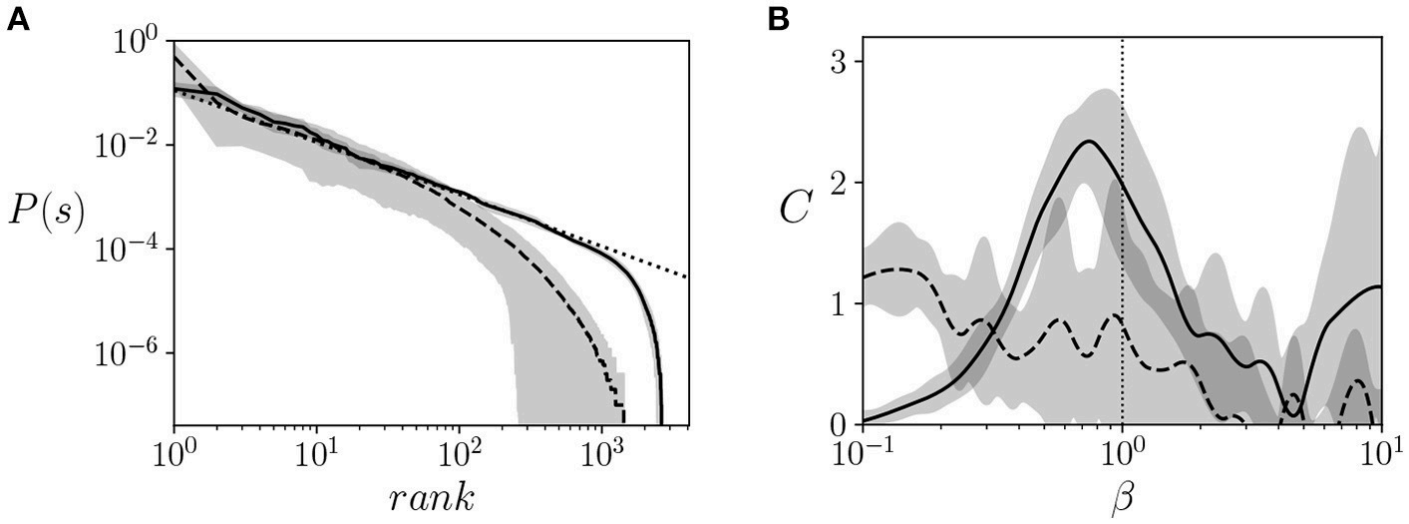
Signatures of criticality. **(A)** Ranked probability distribution function of the neural controllers of trained agents (solid line) vs. a distribution that follows a Zipf's law, (i.e., *P*(σ) = 1/*rank*, dotted line) and the distribution of an agent optimized to solve the task (dashed line). We observe a good agreement between the model and the Zipf's law, suggesting critical scaling. **(B)** Heat capacity $C\left(\sigma_{i}^{\prime }|\sigma \right)$ vs. β of trained agents (solid line), computed using Equation 4 for calculating the entropy and deriving a cubic interpolation of the entropy function with respect to β. The heat capacity of trained agents is compared with the heat capacity of agents tuned to maximize the ability to climb the mountain (dashed lines) The gray areas represent the error bars of the 20 agents for each value of β. The vertical dotted line specifies the value of β = 1 where agents operate during training.

We observed that all agents approximately follow a Zipf's law at β = 1 (Figure 3A) for almost three decades, which is a good agreement for the limited size of the system (note that the possible states of the system are limited to 2^12^ states). All trained agents showed a similar distribution close to Zipf's law. In comparison, agents maximized to solve the task failed to show a distribution that is consistent with Zipf's law.

Secondly, given that another indicator of critical points is the divergence of the heat capacity of the system, we estimated the heat capacity of hidden and motor neurons. From the data generated from the simulation, we can estimate $C\left(\sigma_{i}^{\prime }|\sigma \right)$ by computing entropy $S\left(\sigma_{i}^{\prime }|\sigma \right)$ from Equation 4. We use cubic interpolation for estimating the function of $S\left(\sigma_{i}^{\prime }|\sigma \right)$ with respect to β and for estimating its derivative to compute $C\left(\sigma_{i}^{\prime }|\sigma \right)$. We observe in Figure 3B that the heat capacity peaks at around the operating temperature (i.e., the temperature used during training, β = 1). This, together with the Zipf's distribution, suggests that the system is operating near a critical point. In comparison, agents maximized to solve the task fail to present a clear peak of the heat capacity at a specific temperature, indicating that no significant transitions are taking place.

### 4.2. Behavioral transitions in the parameter space

What is implied when the agent drives its neural controller near a critical point? It should be remarked here that our agents are given no explicit goal. Instead, they only tend toward behavioral patterns that maximize the heat capacity of their neurons, independently of whether this behavior enables them to reach the top of the mountain or not (in fact, only 12 of the 20 trained agents are able to climb to the top of the mountain). In relation to this, we explore the effects of transiting the critical point by observing the different behavioral modes of the agent in the parameter space. The behavior of the car can be described just by the position *x* and the speed *v* at different moments of time.

**Figure 4:**
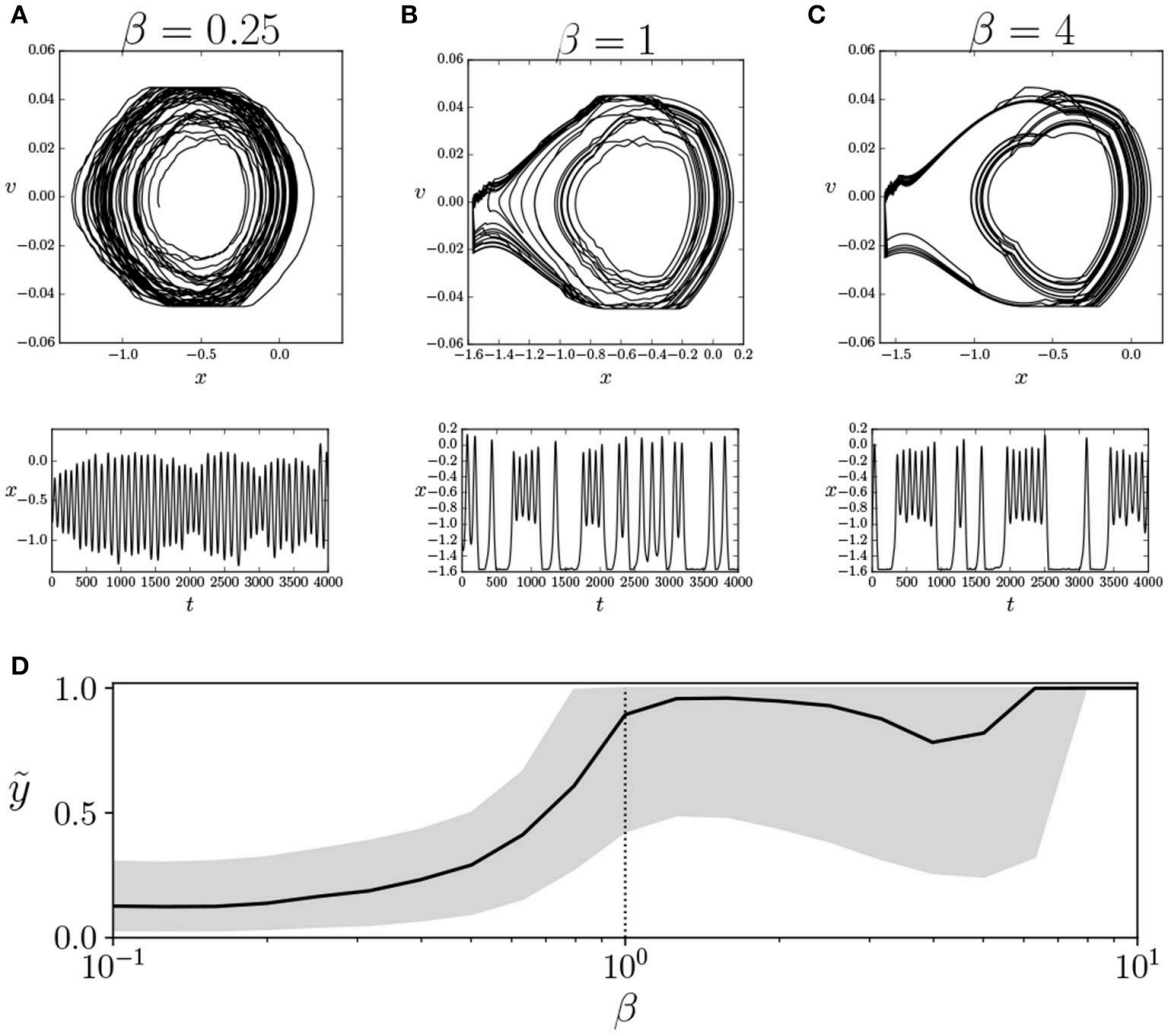
Transition in behavioral regime of the agent. We show the behavior of an agent for an interval of 4, 000 steps with values of β of 0.25 **(A)**, 1 **(B)**, and 4 **(C)**, depicting the trajectories of the car in its phase space (*x* *vs*. *v*, top) and the evolution of the values of *x* (bottom). We observe that β = 1 is a transition point between two modes of behavior. **(D)** Median vertical position of the car ỹ (solid line) and its upper and lower quartiles (gray area). We observe a transition near β = 1 where the agent reaches the top of the mountain. Similar transitions are identified in 12 of the 20 simulated agents. The vertical dotted line specifies the value of β = 1 where the agents operate during training.

In Figures 4A–C, we can observe the behavior of the car for β = {0.25, 1, 4} for a specific agent, for an interval of 4, 000 simulation steps, after an initial run of 10, 000 steps to remove the initial transient. In this particular example, there is an asymmetry in the behavior of the car, which only climbs the left mountain. This asymmetry is provoked by the sign of the offset value *h*_*i*_ of motor units. If we compute the median and quartile values of *y* at the trial for each value of β (Figure 4D) we observe that, slightly below the operating temperature, there is a transition from not being able to reach the top of the mountains to those that are able to do so. Moreover, in all agents that are able to reach the top of the mountain, the results are similar. Out of the agents that are not able to reach the top, five display similar transitions in the median value of height *y* and the median absolute velocity *v* of the car. The remaining three agents fail to show a transition in median values of basic behavioral variables, although this does not preclude the possibility of another type of less evident behavioral transition.

What has changed in this behavioral transition? We are interested in knowing how these behavioral regimes are qualitatively different. We explore this issue by using information theory to characterize how different variables of the agent interact at different points of the parameter space. Specifically, we are interested in the relation between sensor, hidden, and motor neurons, which determines the behavior of the agent in its environment.

**Figure 5:**
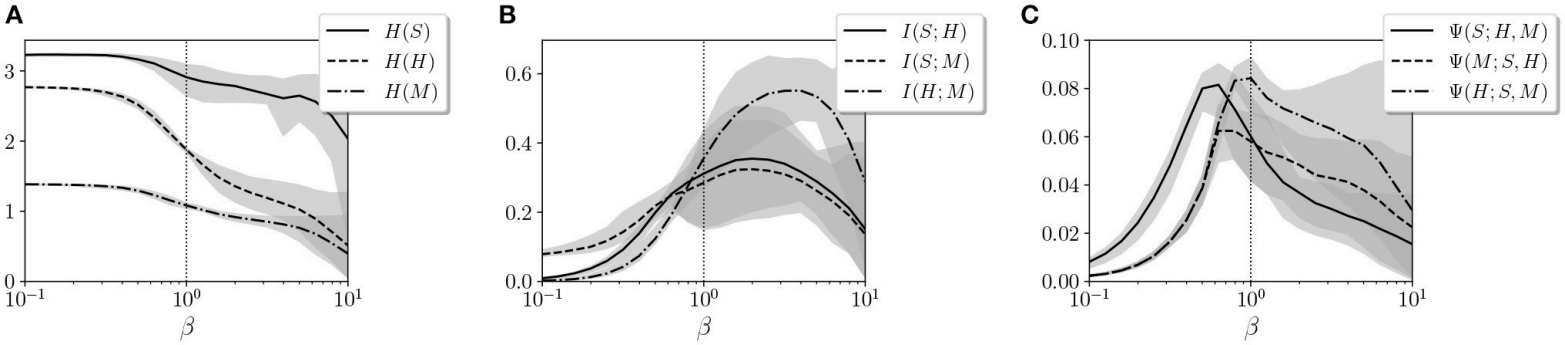
Values of **(A)** entropy, **(B)** mutual information, and **(C)** synergistic information for variables *S*, *H*, and *M*. The gray areas represent the error bars of the 20 agents for each value of β. The vertical dotted line specifies the value of β = 1 where the agents operate during training.

Are agents merely reactive to sensory inputs or is there a more complex interplay between sensor, hidden, and motor units? In order to answer this, we characterize the interaction between variables by using measures from information theory. First, we measured the values of entropy and mutual information between *S*(*X*) and *I*(*X*; *Y*), where

(10) $$S(X)\;=-\sum_{x\in X}P(x)\log(P(x))$$

(11) $$I(X;Y)\;=\sum_{x\in X}\sum_{y\in Y}P(x,y)\log(\frac{P(x,y)}{P(x)P(y)})$$

and *X* and *Y* are random discrete variables. Entropy *S*(*X*) measures the amount of information of each variable, whereas mutual information *I*(*X, Y*) measures the amount of information that overlaps between two variables due to their mutual dependence. In Figures 5A,B, we observe the entropy and mutual information for the variables *S*, *H*, and *M* for different values of β.

By defining *S*, *H*, and *M* as the joint distributions of sensor, hidden, and motor neurons, respectively, we analyze how information is distributed among the three groups of variables. We observe how the entropy of the hidden neuron *H* decreases significantly in the transition around β = 1. Similarly, all values of mutual information, especially between motors *M* and sensors *S*, increase around β = 1. This suggests a transition from independent variables showing unconstrained information to highly correlated variables with high mutual dependencies.

We suspect that it is just in this transition point where an agent can maximize its interactive capacities, combining integration and segregation between variables. To check this, we use information decomposition (Timme et al., 2014) to compute synergies between variables *X*_1_ and *X*_2_ with respect to a third one *Y*, defined as

(12) $$\Psi (Y;X_{1},X_{2})=I(Y;X_{1},X_{2})-I(Y;X_{1})-I(Y;X_{2})+I_{min}(Y;X_{1},X_{2})$$

where *I*_*min*_(*Y*; *X*_1_, *X*_2_), defined in Williams and Beer (2011), is the redundant information that *X*_1_ and *X*_2_ share about *Y*. The resulting synergy Ψ(*Y*; *X*_1_, *X*_2_) is able to capture information about *Y* that is not available from either *X*_1_ and *X*_2_ alone but from their interaction (the classical example is the relation between the output and inputs of an XOR gate). In other words, the intention is to measure how much information emerges from the interaction between variables instead of being contained in the variables alone.

As we observe in Figure 5C, the synergy Ψ(*S*; *H, M*) between motor and hidden neurons about sensor information and the synergy Ψ(*M*; *S, H*) between sensor and hidden neurons about sensor information peak at values of β lower than 1, while the synergies of motor neurons with sensor neurons, Ψ(*H*; *S, M*) peak at larger values of β. Since the environment of the agent is completely deterministic, it seems adequate that larger values of β (i.e., less random behavior) more effectively transmit information from sensors to motors, while maximum interaction between hidden and other neurons occurs at a point with a lower β.

In conclusion, we propose a learning model that is designed to drive an embodied agent close to critical points in its parameter space, poising both the neural controller and the behavioral patterns of the agent near a transition point between qualitatively different regimes of operation. In the case of the neural controller, we find that the Boltzmann machine shows its peak capacity at a point around β = 1. Moreover, by measuring entropy and mutual information between groups of neural units, we find that the agent is poised at a transition point between a regime with high entropy but low coordination between units and a regime of high mutual entropy but low entropy. By analyzing the synergistic interaction between sensor, hidden, and motor units of the system, we find that interactions between groups of neural units are maximized for the operating temperature (although synergistic measures need to be taken into account carefully, since there is an ongoing debate around their formulation, Olbrich et al., 2015). We find a transition at a point slightly under β = 1, which also coincides with a point of transition between behavioral regimes in 12 of the 20 agents. These results suggest that the system may be exploiting a critical point for maximizing the interaction between the components of its neural controller, its sensory input, and its motor behavior.

## 5. Discussion

The rule described here has some similarities and differences with respect to other work on maximizing information quantities in neural networks. Several measures have recently been introduced and have been demonstrated to be viable and powerful tools to express principles for driving autonomous systems. They are measures that are independent of the specific realization and domain invariants. We highlight, for example, predictive information measures (also called excess entropy or effective measure complexity) or methods that maximize entropy reinforcement learning (optimizing policies that maximize both the expected return and the expected entropy of the policy). With respect to the above mentioned measures, our idea differs in some aspects. On the one hand, predictive information is applied at the behavioral level of the whole system (Martius et al., 2013), whereas the learning rule that we propose is defined at the neuronal level (similar to local learning principles like the Hebb rule). Although our rule acts on the internal level, it is linked to information theoretic quantities on the behavioral level. On the other hand, the basic goal in conventional reinforcement learning algorithms is to maximize the expected sum of rewards combined with a more general maximum entropy objective (Ziebart et al., 2008). By contrast, in our method there is no reward that is maximized. Though such algorithms have been successfully used in a number of approaches, our method does not seek to optimize future rewards or maximize behaviors.

At this point, we can reflect on our original questions. Why do biological systems behave near criticality? What are the benefits for a biological system to move toward this special type of point? Also, more importantly, how can our learning model help answer these questions? By reviewing the relevant literature, one finds that interpretations of criticality are too speculative in general. For example, Beggs (2008) hypothesizes that neural systems that operate at a critical point can optimize information processing and its computational power. Mora and Bialek (2011) discuss the experimental evidence of criticality in a wide variety of systems and propose that criticality could provide better defense mechanisms against predators (in animals), gain selectivity in response to stimuli (in auditory systems, or improved mechanisms to anticipate attacks (in immunological systems). Nevertheless, the reasoning that gives support to these hypotheses is based more on generic suggestions than on scientifically rigorous statements. More detailed analyses are needed to test speculations, and our opinion is that conceptual models of embodied criticality in natural systems can usefully demonstrate how transition points in the parameter space of behavioral regimes can be found and exploited to obtain functional advantages such as those mentioned above. For this purpose, rather than citing specific biological instances of critical phenomena, we used an abstract framework for driving embodied agents to critical points. Our model approaches a critical point as well but remains at the disordered phase. Similar phenomena have been observed in biological neural networks (Priesemann et al., 2014). It has been proposed that information flow, generally, peaks on the disordered side of critical phase transitions (Barnett et al., 2013). More detailed studies of general mechanisms that drive a system to criticality may help shed light on these issues.

From the results obtained, what would be the main advantages of using an abstract model to study criticality? On the one hand, criticality generally appears to be entangled with other capabilities that are developed by biological systems, and interpretations about the advantages of criticality typically refer to tangible benefits for the system (e.g., at an evolutionary level, as the source of a new range of capabilities or better mechanisms for surviving in open environments, etc.); it is difficult to distinguish whether criticality is the cause or the consequence of such effects. On the other hand, we believe that the use of conceptual models such as those presented here allows a more intriguing hypothesis to be tested. For example, our general mechanism that drives an embodied neural controller to criticality has the potential to capture the contribution of criticality “by itself” to the behavior of adaptive agents in different scenarios, as well as the relationship between criticality and other biological and cognitive phenomena. Furthermore, the present model could be implemented in more complex embodied setups, for example, involving specific tasks of adaptive behavior that add environmental constraints (e.g., exploration, decision-making, categorical perception) or biological requirements (e.g., an internal metabolism or other biological drives such as hunger or thirst), and it could be used to observe how compliance with these biological and cognitive requirements interplays with the drive toward critical points in the neural controller of the agent. We could, thus, explore how criticality can contribute to the capabilities observed in natural organisms.

Finally, one of the most important conclusions we highlight is that systems at critical points can solve problems for which they were not programmed. This approach can be further linked to the analysis of particular features in animal behavior that are commonly interpreted without assuming a necessary pragmatic perspective of analysis. For example, the role of “play” in humans and other species. We observe certain similarities in the behavior of the developed embodied agent and the notion of play. In general, it is assumed that “solving a problem” is “being able to find a solution.” In computational views of cognition, this requires handling representations of the world between which there is a configuration (the one in which the objective is reached) that the system must find. On the contrary, “play” is precisely not a problem requiring a solution. “Play” does not intend to solve a specific problem. Over time, “play” self-structures processes that are governed by the dialectics of expansion and contraction of possibilities. Its freedom lies in the capability of players to acquire and create novel nonarbitrary constraints during the processes involved (Di Paolo et al., 2010). We think that this may be a good metaphor for how the Mountain Car agent reaches the problem solution.

There are also other studies in the field of “play” that relate creativity, intrinsic motivations, and maximum entropy measurements. For example, Schmidhuber (2010) addresses the problem of how to model aspects of human player behavior that are not explained by either rational or goal-driven decision making behavior and without extrinsic reinforcement such as game score. The author focuses on analyzing the relationship between intrinsic motivation and creativity based on maximizing intrinsic reward for the active creation and the discovery of surprising patterns. In other exploratory studies (Guckelsberger et al., 2017), it is found that metrics such as empowerment can be useful to create specific models of intrinsic motivation in game design. Empowerment (Klyubin et al., 2005, 2008) quantifies the capacity of the agent to influence the world in a way that this influence is perceivable via the agent's sensors. These types of analyses (Roohi et al., 2018) identify the correlations between empowerment and challenge, attention, or engagement, by hypothesizing that maximum entropy measurements can also be used to create support tools for game designers.

In conclusion, we present, here, a model that does not address any particular task but solves a problem. It is interesting to note that it seems to exhibit intrinsic motivations but without being externally imposed, since its behavior is reduced to exploiting the criticality regime in which the system operates. Until now, the traditional study of criticality in living systems has rested on largely speculative grounds. The study of formal models and the increasing amount of high quality data together with advances in statistical mechanics models will make it possible to link experimental evidence and data-driven models with general conceptual models, paving the way for a rigorous exploration of the governing that lie behind the behavior of biological organisms in complex environments.

## Author contributions

MA conceived and conducted the experiments. MA and MB analyzed the results and wrote the manuscript.

### Conflict of interest statement

The authors declare that the research was conducted in the absence of any commercial or financial relationships that could be construed as a potential conflict of interest.
